# Participatory improvement of a template for informed consent documents in biobank research - study results and methodological reflections

**DOI:** 10.1186/s12910-017-0232-7

**Published:** 2017-12-20

**Authors:** Sabine Bossert, Hannes Kahrass, Ulrike Heinemeyer, Jana Prokein, Daniel Strech

**Affiliations:** 1 Institute for History, Ethics and Philosophy of Medicine, Hannover Medical School, Carl-Neuberg-Str. 1, 30625 Hannover, Germany; 2 MEDIAN Centre for Behavioural Medicine, Bad Pyrmont, Germany; 3 Hannover Unified Biobank, Hannover Medical School, Hannover, Germany

**Keywords:** Participatory improvement of informed consent, Informed consent, Patient and public engagement, Focus group interviews, Methodology

## Abstract

**Background:**

For valid informed consent, it is crucial that patients or research participants fully understand all that their consent entails. Testing and revising informed consent documents with the assistance of their addressees can improve their understandability. In this study we aimed at further developing a method for testing and improving informed consent documents with regard to readability and test-readers’ understanding and reactions.

**Methods:**

We tested, revised, and retested template informed consent documents for biobank research by means of 11 focus group interviews with members from the documents’ target population. For the analysis of focus group excerpts we used qualitative content analysis. Revisions were made based on focus group feedback in an iterative process.

**Results:**

Focus group participants gave substantial feedback on the original and on the revised version of the tested documents. Revisions included adding and clarifying explanations, including an info-box summarizing the main points of the text and an illustrative graphic.

**Conclusion:**

Our results indicate positive effects on the tested and revised informed consent documents in regard to general readability and test-readers’ understanding and reactions. Participatory methods for improving informed consent should be more often applied and further evaluated for both, medical interventions and clinical research. Particular conceptual and methodological challenges need to be addressed in the future.

**Electronic supplementary material:**

The online version of this article (10.1186/s12910-017-0232-7) contains supplementary material, which is available to authorized users.

## Background

In human subject research as well as in clinical care, informed consent (IC) is considered an ethical and legal requirement, supporting the protection of participants’ and patients’ rights and maintaining public trust [1–3]. Although there are several well-known limits to informed consent [4, 5], its general importance remains mainly uncontested. For valid informed consent, it is crucial that prospective research participants understand as well as possible all that their consent entails [6]. However, clearly explaining all relevant information for IC has proven to be a major challenge [7]. Challenges include the complexity and amount of information [8] and the readability and understandability of IC documents [7, 9, 10].

Usually, prospective research participants or patients are presented with written information about the planned medical intervention or the research project – mostly accompanied by verbal information by the responsible health-care professional or researcher. Although the importance of written information for valid IC seems to be uncontested, it has been found weak in truly informing prospective research participants [7, 11]. The weaknesses of written information may be one reason that some authors focus on other additional or even alternative ways to inform study participants [12, 13]. However, in a systematic review of interventions aiming to increase understanding in IC, Nishimura et al. show that enhanced consent forms were amongst the most effective [14]. This indicates that, irrespective of other improvements to the consent process, it is worth the effort to carefully test and improve IC documents according to test-readers’ feedback.

As several literature reviews show, a growing number of mainly quantitative studies, including randomized controlled trials, have been conducted specifically to assess understanding in IC in clinical research-settings [7, 14–17]. These studies measure, for example, whether participants understand the purpose and the risks of the research explained in the IC documents, whether they actually grasp the meaning of randomisation, or whether they understand that their participation is voluntary and that they have the right to withdraw. These quantitative approaches are particularly suitable for the systematic assessment of research participants’ actual understanding and memory of certain pieces of information. However, they teach us little about how to solve understanding problems in a way that addresses the requirements of the IC documents’ target population.

In the field of health information, decision aids, and patient information leaflets complementary approaches have been developed to both assess and improve understanding of written information: “User testings” have been applied to written information about various topics [18–21], including patient information documents for clinical trials [22–24]. User testings employ semi-structured individual interviews to not only assess test-readers’ understanding, but also to analyse the findability of different pieces of information, reasons for understanding problems, and test-readers’ general feedback on the informational documents. These findings are used to revise and retest the documents in an iterative process until understanding of all main aspects of the tested documents is achieved.

Although most user testings conduct individual interviews, in the evaluation of written health information and decision aids, focus group interviews have also been used [25, 26]. Focus groups enable participants to comment on each other’s statements, to clear up misunderstandings amongst themselves, and to discuss complex and divisive issues. This allows the assessment of the relative relevance of different feedback – e.g. when participants put their own feedback into perspective after comments by other participants – and the identification of contrasting views, as well as the underlying rationales – e.g. when participants discuss particular issues amongst themselves and give reasons for their differing opinions. These insights from focus groups can facilitate understanding the nature of test-readers’ opinions towards the tested documents. Hence, their requirements and special needs can be addressed more effectively in the revision process.

Additionally, focus groups have been argued to be a suitable means for assessing test-readers’ general perceptions of a given document, including emotional responses to certain issues [26]. This information can help to further improve the readability of information documents, e.g. by explaining emotionally-charged pieces of information and by avoiding ambiguous formulations.

To our knowledge, there has been no attempt to test and improve documents used in the informed consent process using focus groups. Furthermore, prior studies using focus groups did not aim to assess systematically the opportunities and challenges faced by focus group–informed revision of IC documents. In addition, prior focus group studies did not report how the tested documents were revised or whether they were re-evaluated after revision. In this study we aim at further developing the existing methods for participatory improvement of IC documents with regard to (i) assessing their readability, (ii) evaluating readers’ understanding and reactions, (iii) using test results to revise the IC document, and (iv) re-evaluating the revised IC document. For this purpose, we will first describe in detail the methods we used for testing, revising and retesting exemplary IC documents for biobank research; secondly, we will present data and results from this focus group-study; and thirdly critically reflect our methods and results.

## Methods

### Tested informed consent documents

In this study we worked with template IC documents addressing broad consent in biobank research. The template was published in 2013 by a working group of the umbrella organisation for German research ethics committees (AKMEK, Arbeitskreis Medizinischer Ethikkommissionen) [27]. Several expert groups (biobank chairs, members of Research Ethics Committees, experts in data protection, bioethics, and law, and industry representatives) were involved in the development of these IC documents, but no patient or public representatives. The template was particularly suitable for our study, as broad consent for biobank research concerns both patients and healthy people. Research biobanks aim to collect and store human biological samples and related data for an indefinite period of time, ultimately to use samples and data for a broad set of research questions. The tested IC documents include written information for prospective biobank donors as well as the actual consent form.

### Methods for testing IC documents and assessing test-readers’ reactions

**Fig. 1:**
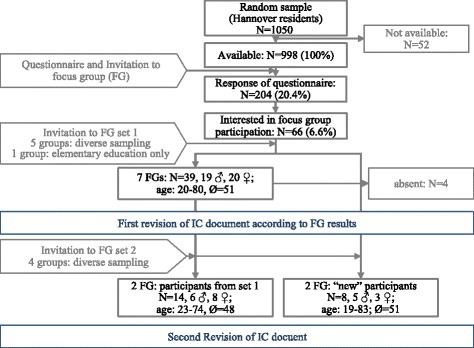
Course of the project

To assess test-readers’ perception of the provided IC documents, we conducted a series of 11 focus groups containing an average of 5–6 residents from an urban region in Germany (Hannover). The first seven focus groups discussed the original IC documents. After analysing audiotapes of these groups and revising the IC documents according to the results, we conducted a second set of four focus groups discussing the revised version of IC documents. Two groups in the second set were conducted with new and two with repeat participants (for an explanation of focus group composition, see below). After analysing audiotapes of this second set of focus groups the documents were again revised accordingly. The course of the project is depicted in Fig. 1. The project was approved by Hannover Medical School’s local research ethics committee (No. 6689–2014). Written informed consent was obtained from all focus group participants.

#### Recruitment and selection of focus group participants

Focus group participants were recruited through a postal survey of a random sample of 1050 Hannover residents. The tested IC documents addressed the general public instead of a particular group of patients. Hence, a sample of the general public best reflects the documents’ target population. All addressees were invited to volunteer for focus group participation. In addition to the focus group invitation, we dispatched a short questionnaire to survey respondents’ knowledge of and attitudes towards biobanks. Respondents’ sociodemographic data (age, gender and school education) was used to enable diverse composition of focus groups. Survey results on knowledge of and attitudes towards biobanks will be published elsewhere. To gather information for the design of future similar projects, we tested three different incentives for focus group recruitment, each offered to 350 addressees: €30, €60 or a taxi voucher to travel to and from the focus group. Response rates for the three recruitment groups were compared. However, in the end, all actual focus group participants were given the highest compensation, €60.

From the pool of volunteers, the actual focus group participants were selected by stratified random sample. We used sex (male/female), age (lower/higher than median) and school education (elementary/middle/higher education) as stratification variables, to ensure each group had a diverse composition. For just one group, we decided to include only elementary-educated participants, to give this sub-group the opportunity to freely express their criticisms of the tested IC documents.

#### Conduct and analysis of focus groups

All 11 focus groups (7 discussing the original and 4 discussing the revised version of IC documents) were semi-structured by an interview guide. In the first four groups, participants were given time to read the IC documents just before the focus group started. All other participants received the documents about a week before the focus group, to read at their leisure. The interview guide was pre-tested in the first focus group in June 2015 and slightly revised afterwards. Because there were only minor adjustments to the guide, and to avoid losing the group’s insightful feedback, this group was included in the analysis. The revised interview guide covered seven topics, which are listed in Table 1. In June/July 2015 six more focus groups were conducted with this final version of the interview guide. After this first set of seven focus groups, the IC documents were revised according to test-readers’ feedback for the first time.

**Table 1:** Focus group interview guide

Topic	Questions – focus groups: set one and two
First impression of text	Free description; no further guiding questions
Knowledge and Understanding	− What is the central message of the text?
	− How understandable is the text?
	− Which topics did you find difficult to understand?
Coverage of relevant information	*Please imagine you had to decide whether or not to donate to a biobank.*
	− How helpful is the text for your decision?
	− What additional information would you like to have?
	− What questions remain?
Readability and Structure	− How easy or difficult was it for you to read the text?
	− What do you think about the length of the text?
	− How do you assess the structure of the text?
	− Do the headings of each paragraph properly describe their content?
Trust/credibility	− Do you trust the information given in the text?
	− What makes you trust/distrust certain pieces of information?
Suggestions for revision	− What suggestions do you have to improve the text?
Additional feedback	Open question; no further guiding questions

In February/March 2016, after the first revision, participants from the first set of seven focus groups were invited to participate in two additional focus groups (of eight and six participants respectively). They were asked to comment on the quality of the revised documents compared to the original versions. In addition, another two focus groups (four participants each) were conducted with new participants who had not seen the original version of IC documents. For this second set of four focus groups (groups 8 to 11) an interview guide (Table 1) with one additional section was used: assessment of certain changes to the original documents. Additionally, the wording of the introduction and of some questions was adjusted to account for the different setting of the second set of focus groups.

All focus groups were facilitated by one of the authors (SB or HK), assisted by a second person (UH, DS or JP), and audio-recorded for analysis. Detailed excerpts were made from the tapes by carefully listening to the whole tapes and paraphrasing all relevant content. Especially relevant statements were transcribed verbatim. For an in-depth analysis of a sub-sample, four tapes (focus groups 1, 2, 5 and 6 from the first set of seven focus groups, each between 00:57 and 1:08 h) were fully transcribed. In addition, participants’ and facilitators’ notes taken during focus groups were analysed. The analysis of excerpts, transcripts, and notes focused on synthesizing participants’ criticisms and suggestions for improving the IC documents’ quality, as well as the systematic identification of understanding difficulties and emotional responses regarding certain topics.

After the first set of seven focus groups, a category scheme was developed by means of qualitative content analysis: we grouped test-readers’ statements into categories according to the subject they dealt with. Further, we combined similar or contrary comments on the same issues into first- and second-order sub-categories. The same category scheme was used to analyse the last four focus groups. Only one additional main category was added, to capture participants’ comments on the changes made to the original IC documents. Based on the results of the analysis, the template IC documents were revised after the first set of seven focus groups, and again slightly adapted after the second set of four focus groups.

### Revision of IC documents

For the revision of the original IC documents we used the sub-categories identified in the qualitative content analysis. To ensure transparent and systematic revision, we developed a traffic-light system to mark our changes in the original document (see Table 2). Colour marking was performed to help track our changes and to distinguish at a glance the degree to which changes were directly based on test-readers’ feedback. In addition, we assigned a distinct code to every single piece of feedback, and noted for every change we made to the original document to which feedback it related. Also, we documented how we addressed each piece of feedback. Where we did not address a particular suggestion, we gave reasons for our decisions. The revised IC documents were discussed by all authors after each revision.

**Table 2:** Traffic-light system to mark changes in original IC documents

Colour^a^	Description of changes
Green	Changes directly based on specific remarks from focus group participants; remarks had to come from at least two participants, preferably from different focus groups and no opposing views from other participants had been expressed. Example: *“First sentence in third paragraph should be divided into two shorter sentences.”*
Amber	Changes directly based on specific remarks from focus group participants; remarks from single participants or disagreement among different participants. Example: *“I would prefer all headings for sub-paragraphs being statements instead of questions.” – “I disagree. I think, all headings should be put as questions.”.* Also, changes which were based on unspecific but unambiguous feedback from focus group participants. Example: *“Paragraph eight is very difficult to understand and should be revised.”*
Red	Changes which were not directly indicated by test-readers but which were made to keep the style of the IC documents consistent. Example: *“The term “Body-materials” in line 15 sounds strange to me” – The term was substituted by the more common term “Biomaterials” not only in line 15 but in the whole document.* Or, changes which were made to address test-readers’ general concerns, misunderstandings or emotional reactions. Example: *There seemed to be a “diagnostic misconception”,* e.g. *some test-readers supposed that donated biomaterials would be used for a complete genetic “check-up”. The respective paragraphs in the IC document were revised to prevent this misunderstanding.*

^a^Decisions between colours were made by the authors directly involved in drafting the revised IC documents (UH, SB, DS). Colour markings are not completely distinctive as some paragraphs required a combination of different kinds of changes, e.g. changes directly based on test-readers’ feedback (green) and more general changes (red) at the same time. However, the colour system was one measure we took to make revisions as transparent as possible

## Results

### Response analysis

In total, 66 of 204 survey respondents volunteered for focus group interviews (see Fig. 1). Of these, 47 participated in the two sets of focus groups: 42 were invited and 39 actually participated in the first set; 22 were invited and participated in the second set (14 participants from the first set and eight new participants). Table 3 shows the characteristics of focus group participants relative to survey respondents as a whole. Focus groups were equally attended by male and female participants; all age-groups as well as all levels of school education were represented. However, individuals with higher education were significantly over-represented amongst survey respondents, as well as amongst focus group participants. There was no significant difference in response rates according to recruitment incentive (see Table 4).

**Table 3:** Characteristics of survey-respondents and focus group (FG) participants

		survey respondents		respondents interested in FG		actual FG participants (rounds 1 and 2)	
Item		valid %^1^	n	valid %^2^	n	valid %^3^	n
sex	male	41.6	(84)	50.8	(33)	51.1	(24)
	female	58.4	(118)	49.2	(32)	48.9	(23)
age	18–29 years	15.9	(32)	17.2	(11)	21.3	(10)
	30–39 years	9.5	(19)	6.3	(4)	4.3	(2)
	40–49 years	16.9	(34)	14.1	(9)	19.1	(9)
	50–59 years	22.9	(46)	31.3	(20)	25.5	(12)
	60–69 years	11.4	(23)	9.4	(6)	10.6	(5)
	70–79 years	16.9	(34)	17.2	(11)	14.9	(7)
	80 or older	6.5	(13)	4.7	(3)	4.3	(2)
school education	low	13.9	(28)	7.7	(5)	6.4	(3)
	middle	23.4	(47)	21.5	(14)	21.3	(10)
	high	57.7	(116)	66.2	(43)	68.1	(32)
	“other”	5.0	(10)	4.6	(3)	4.3	(2)
ever worked in health-care sector?	yes	20.5	(159)	28.1	(18)	28.3	(13)
	no	79.5	(41)	71.9	(46)	71.7	(33)
ever participated in research project (medicine or other)?	yes	15.4	(31)	28.1	(18)	25.5	(12)
	no	84.6	(170)	71.9	(46)	74.5	(35)
nationality	only German	92.1	(187)	96.9	(63)	97.9	(46)
	German and other	1.5	(3)	1.5	(1)	2.1	(1)
	only other	6.4	(13)	1.5	(1)	0.0	(0)

^1^share of missing values between 0.5% and 2.0% of *N* = 204 respondents

^2^share of missing values between 1.5% and 3% of *N* = 66 persons interested in focus group participation

^3^share of missing values between 0% and 2% of *N* = 47 focus group participants

**Table 4:** Response rates by recruitment-groups

	n (persons available)	response rate – survey		response rate – focus group	
		% of recruitment-group^a^	n	% of recruitment-group^b^	n
group 1: €60	332	21.7	(72)	7.2	(24)
group 2: €30	331	16.9	(56)	6.0	(20)
group 3: free taxi	335	22.7	(76)	6.6	(22)
Total		20.4	(204)	6.6	(66)

^a^Differences in response rates tested by means of chi2-test; χ2 = 3.881, *p* = 0.144 (two-sided)

^b^Differences in response rates tested by means of chi2-test; χ2 = 0.380, *p* = 0.872 (two-sided)

### Category scheme for focus group-feedback

The category scheme developed after the first set of seven focus groups consists of eight primary categories: 1) Text length, 2) structure, 3) style/language, 4) understandability/clarity, 5) comprehensiveness, 6) trust in the information provided, 7) emotional reactions, 8) others. The same category scheme was used for the analysis of the second set of four focus groups. Only one additional primary category, “assessment of changes to original text”, was added. Additional file 1: Table S1 (online supplement 1) presents the complete category scheme, including first- and second-order sub-categories. In the following we describe core results from the focus group feedback.

### Focus group feedback on the original IC documents

Participants’ comments on the tested IC documents were mixed. While overall they regarded the text to be quite understandable and easy to read, they gave detailed feedback on some aspects they thought needed improvement. These included the length of the text, its structure, style and language (citations 1–3 in Table 5). Participants also mentioned certain pieces of information they found obscure, such as “long-term storage” (citation 4) or “pseudonymisation” (citation 5).

**Table 5:** Citations from focus group excerpts

Topic	No. of Citation	Selection of focus group (FG) feedback^a^
*Feedback on formal aspects of the text*		
Length	Cit. 1	*“The text is very long which makes it boring to read.” (excerpt FG7)*
Structure	Cit. 2	*“The main purpose of the text is to recruit biobank donors. This should be made clear right at the beginning of the text.” (excerpt FG4)*
Style	Cit. 3	*“In almost all paragraphs, the text uses many nouns and some sentences are way too long.” (excerpt FG2)*
*Issues that were difficult to understand*		
Long-term storage	Cit. 4	*“What does ‘long-term storage of data and biomaterials’ mean?” (excerpt FG3)*
Pseudonymisation	Cit. 5	*“I did not understand the difference between ‘pseudonymisation’ and ‘anonymization’”(excerpt FG6)*
*Concrete suggestions for text-revisions*		
Rephrasing paragraph	Cit. 6	*“The paragraph on potential benefits of biobank research consists of one five-line-long sentence. This paragraph should be revised.” (excerpt FG4)*
Adding illustrations	Cit. 7	*“You should add some figures to illustrate the process of biobank donation and usage of the donated materials for research.” (excerpt FG7)*
*Emotional responses*		
Balanced information	Cit. 8	*“All things considered, the text is surprisingly understandable and the information it gives appear impartial and balanced.” (excerpt FG4)*
Trustworthiness	Cit. 9	*“The text seems very objective and trustworthy. It does not try to influence readers in one or the other direction.” (excerpt FG5)*
Discomforting terms	Cit. 10	*“At first, the term ‘body materials’ scared me. I thought they wanted to rip out parts of my body. Then I realized it’s only blood, urine, and things like that.”(excerpt FG2)*
Discomforting issues	Cit. 11	*“Why do they only give feedback on incidental findings when these are ‘relevant’ to my health? I want to get to know everything about my health. And, I don’t like others to decide what is relevant to my health.”(excerpt FG5)*
Wish for more supervision	Cit. 12	*“Who can guarantee that the researchers using my biomaterials and data do not misuse them for their own purposes? Can I trust the supervising bodies?” (excerpt FG5)*
Phrases causing distrust	Cit. 13	*“The text keeps repeating the phrase ‘we assure you… this, we assure you… that…’. This sounds too much as if they want me to believe everything they say.” (excerpt FG4)*
*Exemplary Misunderstandings*		
Biobank mistaken for “organ-bank”	Cit. 14	*“The text asks me to donate ‘body materials’. Could that include an eye or my heart? So, do they ask for organ donations, too? How can I be sure, they do not take these parts of my body and give them to somebody else?” (excerpt FG2)*
“Diagnostic misconception”	Cit. 15	*“I consider all incidental findings about my health are relevant to my health.” (excerpt FG5)*
*Additional Feedback*		
Use of technical devices/videos to support written information	Cit. 16	*“Couldn’t you use web applications or videos to give extra information on certain topics?* E.g. *one could read the text on a tablet computer and click on topics they are interested in to get more information.” (excerpt FG7)*
Dynamic presentation of information	Cit. 17	*“Perhaps sum up all key aspects at the beginning of the document for everybody to read. Persons, who are interested in the whole document could then read the longer version, too.” (excerpt FG2)*
*Feedback on revised IC documents*		
General approval	Cit. 17	*“For me, this text is very understandable and easy to read.” (excerpt FG11; new participants)*
Added information box	Cit. 18	*“The information box giving the most important points on the first page is very helpful and makes you want to read on.” (excerpt FG9; new participants)*
Added illustration	Cit. 19	*“At first, I did not understand what happens to my biomaterials and data after I give my consent. But thanks to the illustration this is very clear now.” (excerpt FG9; new participants)*
Text-boxes	Cit. 20	*“Most paragraphs are already very short and easy to understand. Additional text boxes which summarise the most important statements are just not necessary.” (excerpt FG11; new participants)*
Longer explanations	Cit. 21	*“Some of the added information are too detailed and could be removed from the actual IC documents; they rather should be served as additional information for especially interested persons.” (excerpt FG8; old participants)*
	Cit. 22	*“I actually understood for the first time what ‘findings relevant to your health’ means; but the new version is just too long for lay people to understand.” (excerpt FG10; old participants)*

^a^Citations are paraphrased for better readability and translated from German into English by one of the authors (SB)

While some feedback addressed more general problems for readability and understandability, participants also made concrete suggestions for the revision of the IC documents, such as rephrasing certain paragraphs (cit. 6) or adding figures and illustrations to the text (cit. 7).

Test-readers also gave feedback on how much they trusted the given information and how they felt about the tested documents in general. Most participants said they trusted the information given, and they felt the documents aimed to give objective and well balanced information (cit. 8, 9). However, some participants indicated that certain terms, e.g. “body-materials” (cit. 10) and topics, e.g. feedback on incidental findings (cit. 11) caused discomfort. Individual participants also expressed general distrust and wished for more supervisory bodies (cit. 12), others found phrases in the tested IC documents caused distrust (cit. 13), and hence should be revised.

Some of the emotional responses and distrust expressed could be addressed by rephrasing the relevant paragraphs in the documents. Other negative reactions seemed to be caused by misunderstandings which could be cleared up by better explaining certain topics (cit. 14, 15).

Several test readers also made additional suggestions for the design of the tested document or for the consent process as a whole – e.g. to use multimedia devices or videos to support written information (cit. 16) or to add a short version with the key aspects at the beginning of the IC documents (cit. 17).

In sum, the results of the first set of focus groups indicated that the original IC documents were performing adequately in terms of completeness and balance of the given information, as well as for understandability. However, some topics were difficult to understand, and participants made suggestions to improve readability by changing the structure and style of the text.

### Revision of IC documents and feedback on the revised versions

**Fig. 2:**
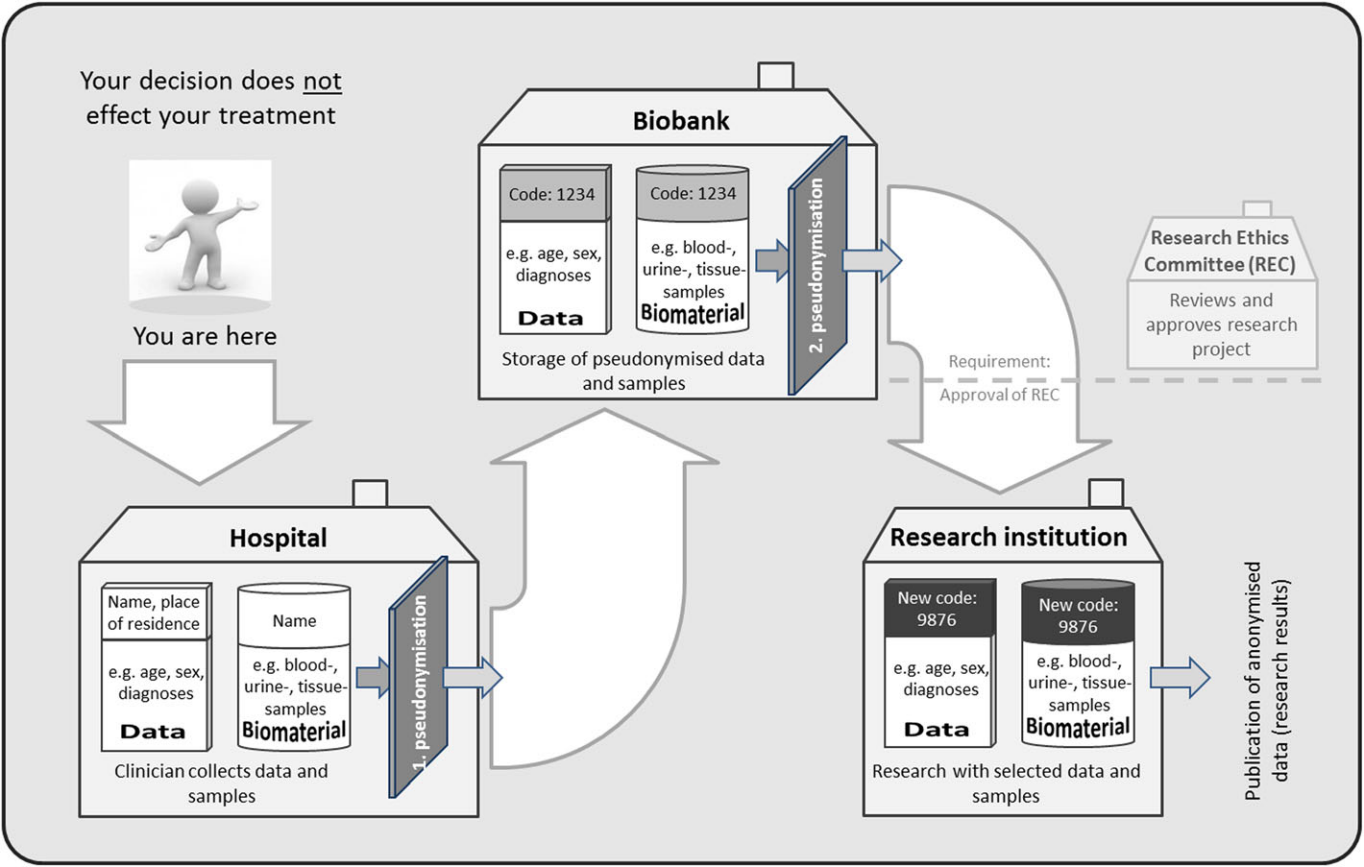
Graphic added to the IC documents after first set of focus groups

To address the test-readers’ feedback, we revised the original IC documents, using the traffic-light colour system described in Table 2 to track the changes we made. Changes to the text included:

- Adding a table of contents;
- Adding a summary of the most important points to the front page, including the general purpose of the IC documents (e.g. recruiting participants for biobank research), the general purpose of biobanks, and participants’ rights (e.g. not to participate, to withdraw at any time, etc.);
- Adding text boxes which summarised key points of each main section of the text (e.g. character and purposes of the biobank, data collection and data storage, participants’ rights, etc.);
- Adding a graphic to illustrate the processes of biobank donation and biobank research (see Fig. 2);
- Making some points more explicit, and explaining certain pieces of information in more detail (e.g. clarifying the concept of “long-term storage”, explaining the concept of “incidental findings relevant to your health” and the possibility of reporting them to participants);
- Shortening some over-long sentences;
- Changing or better explaining technical terms (e.g. “pseudonymisation”, “bio-materials”, “Research Ethics Committee” etc.).

After the first revision, the IC documents were tested in four more focus groups. Feedback from both old and new participants was mostly favourable (cit. 17 in Table 5). Some of the new participants, who had not seen the original documents, specifically praised some of the revisions, without knowing they were new, such as the added information-boxes (cit. 18) and illustration (cit. 19). These elements were also positively rated by repeat participants, who had already read the original version. However, participants in all four focus groups disliked some changes to the original version*.* In particular, some of the added text boxes were perceived to be unnecessary (cit. 20). Also, to clarify certain topics the revised version contained some more-detailed and therefore longer explanations. In a direct comparison of the original (mostly shorter) to the revised (longer) explanations, the feedback was mixed. Although participants acknowledged that the longer version was easier to understand, they thought it included some too-detailed information (cit. 21); the text was already too long to read for most laypeople and therefore they argued to shorten the explanations (cit. 22).

After analysing all focus groups, we again revised the IC documents. The changes we made in the second revision, however, were mainly marginal or partly reversed prior changes to the original document, including:

- Shortening previously revised explanations;
- Rephrasing some over-long sentences;
- Removing unnecessary info boxes (only the box summarising the key points of the whole text was retained).

The final version of the tested IC documents was discussed until all project members agreed that it best reflected test-readers’ feedback.

### Methodological findings and limitations

Aside from evaluating and improving the tested IC documents, we also intended to contribute to the further development methods for participatory improvement of IC documents. Some methodological issues are exemplified and discussed in the following paragraphs:

#### Recruitment and compensation of focus group participants

The IC documents we tested addressed members of the general public, rather than patients living with particular diseases. We therefore recruited focus group participants from a random sample of the general public. For recruitment of focus group participants we used three different amounts of compensation as incentives (€30, €60, free taxi). As there was no significant difference in response rates for the three groups (Table 4), we assume that the amount of compensation promised to addressees did not influence their willingness to participate in the study. It is widely debated whether research participants in qualitative research should be financially compensated [28, 29]. We decided to treat focus group participants as experts, who are usually compensated for each one-time-consultation. Therefore all participants were provided with EUR 60 for each focus group they participated in.

#### Response rate and composition of focus groups

Only 6.6% of invited persons volunteered for focus group participation. This causes concerns regarding potential biases. However, we did not aim for statistical representation of the target population, but rather aimed to identify the whole spectrum of different comments on the tested IC documents. For this purpose, we applied a stratified sampling strategy to achieve a diverse composition of each focus group. Overall, our sampling aim of recruiting a diverse sample of participants was fulfilled: except for one group for only elementary-educated participants, participants of all focus groups represented different age groups, levels of education and personal backgrounds.

#### Number of focus groups

In the first set we conducted seven focus groups. However, after the first three or four focus groups, most feedback did not raise new issues, but merely confirmed feedback from prior groups. We therefore assume that in the first set of focus groups a lower number of groups would have been sufficient to obtain test-readers most important criticism, suggestions and emotional responses. In the second set, four groups – two with former and two with new participants – appeared to be adequate to assess the full spectrum of feedback and to confirm several critical aspects in more than one group.

#### Discussing revised IC documents with new and repeat focus group participants

Many comments in the second set of focus groups were given by both former and new participant groups. However, this was not perceived as redundant. On the contrary, obtaining consistent feedback from both kinds of participants was helpful to confirm some of the changes to the original documents and point out need for additional revisions. While new participants had a fresh perspective and, hence, were able to give information about whether the documents were understandable to potential future biobank participants; repeat participants were asked to validate and approve the changes made according to their feedback in the first set of focus groups. Combining these two pieces of information allowed to better assess the quality of our revisions and of the revised documents as a whole with regard to readability, understandability as well as test-readers’ perception and emotional responses. Hence, we would recommend inviting both, new and repeat participants to discuss the revised version of the tested documents.

#### Reading tested documents in advance vs. at the beginning of focus group

Providing the tested IC documents directly before the focus groups enabled us to obtain participants’ spontaneous reactions to the text. This was especially valuable for assessing their emotional responses. However, based on our content analysis, all project members agreed in the impression that feedback by participants who read the IC documents in advance was more nuanced and more detailed. These participants also seemed more confident to give definite feedback, because they had been able to consider their opinions of the issues. In addition, some had made detailed written notes in their version of the documents, which were used as a complementary source for revisions. Hence, both variations – reading documents in advance and directly before focus groups – have proven advantageous in a complementary manner for testing and revising IC documents.

## Discussion

### Discussion of applied methods

In this study we tested, revised and retested IC documents for use in biobank research by means of focus groups. This discourse-oriented method was chosen to obtain test-readers’ detailed and nuanced feedback and suggestions, as well as to identify possible misconceptions, controversial opinions and emotional reactions to certain topics.

Overall, focus group interviews have proven to be a viable means for the participatory improvement of IC documents. Participants gave valuable feedback for revising the original documents. Like in other “user testings” many comments concerned the application of general rules for clear writing, such as using short sentences, or not using technical terms [e.g. 22, 30]. But participants also gave detailed feedback on certain facts they found hard to understand or about additional information they would like to have for their consent decision. In a recent Delphi procedure, Beskow and colleagues identified a set of key points prospective biobank donors must grasp before being able to give valid consent [31]. This includes: purpose of data and specimen collection, necessary procedures, duration of data and specimen storage, risks, confidentiality protections, benefits and costs, voluntariness, discontinuing participation, whom to contact for questions, Commercialisation of stored material, and handling of new findings with relevance to the subjects’ own health. The points in their list correspond well with the topics about which our test-readers gave most feedback. Hence, their comments addressed essential aspects of the IC documents.

In contrast to individual interviews used by most “user testings” [19–24, 30], the discursive focus group setting also allowed for controversial discussion amongst the participants, which helped reveal contrasting opinions and misunderstandings, e.g. a discussion in one focus group revealed that some test-readers presumed that all donated specimens were analysed by the biobank and, hence, they believed that participants could hope for diagnostic benefits. This belief made them indignant about the formulation in the IC document that “only incidental findings with direct relevance to your health” would be communicated. After identifying this misunderstanding, we were able to clarify the formulation in the IC documents. All findings from the focus groups were used to revise the original IC documents in two rounds. Comments on the revised IC documents by participants of the second set of focus groups, as well as by biobank experts, were mainly positive. This indicates that the revised IC documents were an improvement. However, we have not yet measured by means of a randomized controlled study whether certain indicators for understanding have been improved, and if so to what degree. Such RCT methodology has already been used to evaluate IC quality [14] and could also be applied in further research on the effects of the participatory improvement of IC documents.

### Practical and conceptual challenges

In the course of our study we encountered a set of practical and conceptual challenges which could inform future similar studies, and also indicate the need for further methodological research on the evaluation and improvement of consent documents [32]. Firstly, how can we better distinguish the different dimensions of “understanding” with regard to health care or research-related texts, including IC documents? Research on informed consent in clinical research has attempted to better distinguish between misconceptions, misestimates and optimism [33]. These conceptual dimensions, however, all deal with testing understanding in the above-mentioned paradigm of objective, survey-based IC research. In our study we often dealt with a complementary but more preliminary dimension of understanding, that is, whether a reader has the subjective impression of grasping the text message. Further conceptual and empirical research is needed to assess the theoretical and practical relevance of integrating subjective and objective dimensions of understanding in evaluating and improving health and research-related texts.

Secondly, as mentioned above, the IC documents we tested were designed for informing members of the general public about participating in biobank research. To gain information about this group’s specific needs and potential problems in understanding the IC documents, we invited members of this group to participate in focus groups. However, other IC documents in clinical research or care aim at informing and recruiting other groups – e.g. patients, their relatives or carers. Hence, for effectively testing and improving these IC documents, members of the respective target population should be included. We believe that the results of our study are applicable to other target populations too. Despite several differences between target populations, most of them do not possess medical expert knowledge and can therefore be perceived as lay people. Learning about lay peoples’ perception of the tested IC documents can help improving their quality in the above described manner. However, further empirical research is needed to confirm this perception and to analyse potential differences between patients, research participants, members of the general public and other relevant groups as sources for improving IC documents.

Thirdly, for effectively improving written information according to test-readers’ requirements, revisions need to actually reflect their feedback. However, our test-readers’ opinions did not always indicate obvious changes in the original document. Sometimes their opinions differed or were based on what we interpreted as prevailing misconceptions. Furthermore, some reasonable requirements were hard to meet simultaneously – like the wish for more detailed explanations and the demand for shortening the respective paragraph. Some solutions for this particular Problem – e.g. using videos or electronic IC documents to present information in a dynamic way (cit. 17 and 18 in Table 5) – have already been suggested by our test-readers’ themselves and by the literature on dynamic consent [34]. However, for a transparent revision of tested documents, systematic solutions for how to address ambiguous or contesting kinds of feedback can be addressed in the revision process. Authors of user tests have named three sources for their changes to the original documents: first, feedback from user testing; second, best practice guidelines in information wording and clear writing; and third, authors’ experiences with writing patient information documents [20, 22–24]. These are indubitably important sources for revisions. But, to our knowledge, there are no detailed reports how exactly revisions were made based on test-readers’ feedback, and how authors dealt with the above outlined challenges. Of course, revised documents should comply with best practice guidelines; how can those responsible for the participatory improvement of IC documents demonstrate that revisions also meet test-readers’ feedback in a systematic and unbiased way?

Finally, the methodology we applied in our study is rather complex and costly and hence might not be feasible in many contexts. E.g., in some cases there might not be enough potential participants for a high number of focus groups. Also, conducting and analysing focus groups takes a lot of time which might not be available. To comply with limited resources, one could e.g. reduce the number of focus groups or the number of participants in each focus group. Other user tests have used individual interviews to test, revise and retest written information [e.g. 20, 22, 23, 24]. This might also make the process less costly and more feasible. However, additional conceptual and empirical research is needed to systematically compare different methods for testing and revising written information and to analyse the respective advantages and disadvantages of each method.

## Conclusion

Although in our study we used IC documents intended to inform prospective biobank donors, we believe our findings are applicable for the participatory improvement of IC documents for other research settings as well as for medical interventions, too. Our results indicate that focus groups are a suitable complement to other methods for participatory text improvement – like the more formal user tests using individual interviews [e.g. 20, 22, 23, 24]. In all settings of participatory text improvement, however, further conceptual and empirical research is needed to identify and address the full set of challenges in assessing and revising texts based on test-readers’ feedback. The clarification of these challenges can contribute to a consolidated conceptual model for the development and improvement of IC documents and other kinds of written information.

## Additional file

Additional file 1: Table S1. Category scheme for test-readers’ feedback on IC documents. Table S1 contains the detailed category scheme developed for content analysis of focus groups. (DOCX 17 kb)

## Abbreviations

AKMEK: Organisation for German research ethics committees (“Arbeitskreis Medizinischer Ethikkommissionen”)
Cit.: Citation from focus groups
IC: Informed consent
