# Supplementary material for: Participatory improvement of a template for informed consent documents in biobank research - study results and methodological reflections
Source: BMC Med Ethics. 2017 Dec 20;18:78. doi: 10.1186/s12910-017-0232-7 (PMC5738718; doi:10.1186/s12910-017-0232-7)
Supplement: Additional file 1: Table S1. — Category scheme for test-readers’ feedback on IC documents. Table S1 contains the detailed category scheme developed for content analysis of focus groups. (DOCX 17 kb) [file 12910_2017_232_MOESM1_ESM.docx]

Table S1: Category scheme for test-readers’ feedback on IC documents

| **Primary categories** | **First-order sub categories** | **Second-order sub categories** |
| --- | --- | --- |
| Length of the text | Overall evaluation | Text too long, general wish for shorter text |
|  |  | Reasonable length |
|  | Suggestions for abbreviation | Exclude certain pieces of information (several concrete suggestions) |
|  |  | Rephrase lengthy paragraphs, omit certain words (several concrete suggestions) |
| Structure | Overall evaluation | Structure partially confusing |
|  | Objectives and central information of the text need to be presented on first page | Rephrase first paragraph to make clear the main objectives |
|  |  | Include box with key information on first page |
|  | Feedback on subheadings | Subheadings helpful to structure the text |
|  |  | Formulation and formatting of subheadings needs to be harmonized |
|  | Feedback on cross references | Cross references are confusing |
|  |  | Cross references helpful for orientation in the text |
|  | Suggestions for formatting and structuring | Insert table of contents |
|  |  | Insert figures to illustrate complex topics |
|  |  | Highlight important pieces of information (e.g. by underlining) |
|  |  | Give important information in bulleted lists instead of running text |
|  |  | several concrete suggestions to insert line breaks or change the position of certain paragraphs |
| Style/language | Overall evaluation | Language overall well-balanced |
|  |  | Different paragraphs use heterogeneous language |
|  | Suggestions to improve style and language | Rephrase long sentences (several concrete suggestions for division of long sentences) |
|  |  | Omit use of subjunctive (several concrete suggestions) |
|  |  | Omit nominalized verbs (several concrete suggestions) |
| Understandability/clarity | Overall evaluation | Text is quite comprehensible |
|  |  | Text incomprehensible to less-educated persons or persons with little knowledge of the German language |
|  |  | Some parts of the text sound like “legal German” and are not comprehensible |
|  | Omit or explain technical terms | (Several concrete suggestions) |
|  | Unclear information needs to be explained in more detail | Duration of storage in biobank |
|  |  | Rules for usage of biomaterials for research |
|  |  | Feedback on incidental findings |
|  |  | Concrete consequences of consent and withdrawal |
|  |  | Who to contact for further queries or withdrawal |
|  | Misunderstandings that have become obvious in focus groups | Confusion of biobank donation with organ donation |
|  |  | “Diagnostic misconception”: assumption that biobank donation would be used for systematic screening for certain diseases |
| Comprehensiveness of given information | Overall evaluation | Information is sufficient to decide on biobank donation |
|  |  | Some additional information could be useful |
|  | Suggestions for additional information | Details on biobanking and its objectives in Germany |
|  |  | Examples of research projects or research questions using biobanks |
|  |  | Who else is invited to donate to the biobank (e.g. all patients at a hospital / a certain selection of patients) |
| Trust in the text and the information provided | Overall evaluation | The text does not exert pressure and allows for a “free decision” |
|  |  | Trust in objective and well-balanced information given by the text |
|  |  | The repeated assurance of the text’s trustworthiness rather causes distrust |
|  |  | Expression of general wish for supervisory authorities in research |
|  | Suspicion of certain information | Doubts about adherence to rules on data security |
|  |  | Doubts about the right to withdraw “without consequences” |
| Emotional reactions | Certain phrases or terms that cause discomfort or are perceived to be inappropriate | (Several examples named, e.g. “body-materials”, “residual materials”) |
|  | Pieces of information that cause discomfort | Feedback only on “relevant” incidental findings: “Who decides what is relevant?” |
|  |  | Storage of biomaterials and data for an indeterminate period of time – “This may carry unforeseen risks.” |
|  |  | Necessity of pseudonymization instead of anonymization |
|  |  | Access to biomaterials and data by foreign research institutions and pharmaceutical companies |
| Additional feedback | Technical terms can be explained in an additional glossary |  |
|  | Present the text on digital devices with hyperlinks for technical terms and complex pieces of information |  |
|  | Present information in videos, or use interactive media instead of written documents |  |
| Additional category for second set of focus groups: | | |
| Assessment of changes to original text | Assessment of new structure (including table of contents and summary of most important points on first page) |  |
|  | Assessment of more detailed explanations |  |
|  | Assessment of added graphic |  |
|  | Further feedback on revisions |  |
